# Neurocognitive outcomes and memory transfer in heart transplantation

**DOI:** 10.21542/gcsp.2025.43

**Published:** 2025-08-30

**Authors:** Patrick Ashinze, Wuraola Salawu, Eniola Akande, Suvam Banerjee, Abdullaah Idris-Agbabiaka, Bethrand Chukwu, Nelson Mafua, Francis Ngirigwa, Tesleem Okeoyo, Stephen Olowookere, Victor Mayowa Adeleye, Joseph Tolulope Olajuwon, Caleb Aboderin, Ayodeji Olasemo, Lukman Abiodun Musa

**Affiliations:** 1 Faculty of Clinical Sciences, University of Ilorin Teaching Hospital, Ilorin, Nigeria; 2 St John’s Hospital, Livingston (NHS Lothian) Scotland, United Kingdom; 3 Burdwan Medical College and Hospital, Department of Health and Family Welfare, Government of West Bengal, India; 4 Georgetown Public hospital, Guyana; 5 Faculty of Clinical Sciences, Madonna University, Anambra, Nigeria; 6 Federal Medical Centre, Asaba, Delta, Nigeria; 7 The Balfour Hospital, NHS Scotland, United Kingdom; 8 Obafemi Awolowo University Teaching Hospital Complex, Ife, Osun state, Nigeria; 9 Department of Community Medicine and Public Health, Ekiti State University Teaching Hospital, Ekiti, Nigeria; 10 Mersey and West Lancashire Teaching Hospital NHS Trust, United Kingdom; 11 Emergency Department, Aberdeen Royal Infirmary, Foresterhill, Aberdeen, Scotland, United Kingdom

## Abstract

Introduction: Cardiac transplantation remains a life-saving intervention for end-stage cardiac disease, substantially improving survival and quality of life. While physiological and immunological challenges, such as graft rejection and immunosuppression, are well-characterized, emerging evidence underscores complex neurocognitive and psychological transformations in recipients. These include debated phenomena such as shifts in memory, behavior, and personality, which challenge conventional paradigms of transplantation outcomes.

Methodology: This scoping review was conducted by performing comprehensive literature searches of databases including PubMed, Scopus, Google Scholar, Cochrane Library, and PsycINFO using MeSH keywords: “heart transplant”, “neurocognitive outcomes”, “cellular memory”, AND “personality transfer”. The timeline spanned 1990 through June 2025. Inclusion criteria: (1) peer-reviewed clinical studies; (2) case reports providing detailed narrative descriptions relevant to memory/personality changes; (3) manuscripts written in English; (4) reports including ≥5 participants/patients. Exclusion criteria: (1) non-transplant cardiac studies; (2) animal research; (3) editorials/opinions/gray literature; (4) non-English manuscripts. Study quality was assessed through manual review of documented accounts alongside corresponding peer-reviewed manuscripts.

Results: This review synthesizes theories, case studies, and mechanistic hypotheses from published reports, exploring neurocognitive trajectories and purported memory-behavioral transfer between donors and recipients. Epigenetic modifications—such as DNA methylation and histone remodeling—are hypothesized to alter gene expression in donor-derived cells, potentially influencing recipient cognition and behavior. Concurrently, immune-brain crosstalk, mediated by cytokines and neuroinflammatory pathways, may exacerbate psychological distress, including identity dissonance and anxiety. Cognitive-behavioral interventions and psychosocial support emerge as critical tools for post-transplant adaptation.

Conclusion: Current evidence on memory transfer remains inconclusive, yet its implications for biological-psychological interconnectedness warrant rigorous interdisciplinary inquiry. By integrating neuroscientific, immunological, and psychological frameworks, future research can elucidate the mechanisms underlying post-transplant neurocognitive phenomena, optimizing therapeutic strategies and patient counseling.

## 1. Introduction

Heart transplantation has been the gold standard for end-stage heart failure, significantly improving survival and quality of life for patients^1^. Advances in surgical techniques, immunosuppressive therapy, and post-transplant care have contributed to a median survival of approximately 12–15 years, with 1-year survival rates exceeding 85% in developed settings^1,2^. Despite these successes, heart transplant recipients often face long-term complications, such as neurocognitive impairments that can impact daily functioning and overall well-being^3^. While initial transplant efforts focused on improving organ viability and managing rejection, attention has increasingly shifted toward understanding the long-term neurological and psychological outcomes, as these factors directly impact the success of transplantation beyond survival alone.

Neurocognitive function is crucial for post-transplant recovery, as cognitive decline has been observed in a subset of recipients. Factors such as perioperative cerebral hypoxia, systemic inflammation, immunosuppressive drug effects, and preexisting comorbidities contribute to cognitive changes following transplantation^4,5^. These alterations may manifest as memory deficits, reduced executive function, and difficulties in attention and processing speed, affecting patient autonomy and mental health. Additionally, neurocognitive impairment has been linked to poor medication adherence, increased hospitalizations, and lower overall quality of life in transplant patients, highlighting the need for early detection and intervention^6^. A comprehensive understanding of these neurocognitive outcomes is essential to improving long-term care strategies and enhancing transplant success beyond mere organ survival.

An intriguing and controversial aspect of post-transplant cognitive changes is the concept of memory and behavioral transfer. While the heart has traditionally been viewed as a mechanical pump, anecdotal reports suggest that some recipients experience personality changes, new preferences, and even memories they associate with their donors^7,8^. These reported changes include alterations in food cravings, shifts in artistic preferences, and even vivid dreams or flashbacks related to donor experiences, raising questions about the role of the heart in memory and consciousness. Theories attempting to explain this phenomenon range from cellular memory and neuropeptide signaling to psychosocial influences and medication effects^9^. However, scientific consensus on this subject remains lacking, with skeptics attributing the changes to psychological adaptation, immunosuppressant-induced neurocognitive alterations, or mere coincidence^10^. Recent research into cellular communication and the potential influence of donor DNA fragments in recipient physiology has further fueled this debate, suggesting that heart transplantation may involve more than just replacing a failing organ.

This review aims to critically examine neurocognitive outcomes following heart transplantation while exploring the emerging debates on memory and behavioral transfer. By synthesizing current evidence from clinical studies, patient case reports, and neuroscientific theories, this article seeks to provide a balanced perspective on the extent and implications of cognitive and behavioral changes post-transplantation. Given the increasing number of heart transplant recipients worldwide, a deeper understanding of these phenomena could shape future transplantation protocols, influence patient counseling strategies, and refine rehabilitative interventions aimed at optimizing cognitive health post-surgery.

## 2. Neurocognitive Outcomes After Heart Transplantation

### 2.1 Cognitive function pre- and post-transplant

Patients with heart failure, particularly those at advanced stages, tend to have baseline cognitive impairments, even before heart transplantation. Such pre-existing deficits are caused by a multifactorial interaction of mechanisms, which include reduced cerebral oxygenation, systemic inflammatory responses, and the direct impact of cardiac dysfunction on brain function. The most common cognitive domains affected include executive function, attention, memory, and psychomotor speed^11^. The extent of these deficits can vary considerably, depending on factors such as the stage of heart failure, comorbid conditions, and other patient-specific factors^11,12^. Specifically, reduced cardiac output in heart failure can result in compromised cerebral perfusion, directly impacting neural function. Chronic inflammation, which is frequently associated with heart failure, can also lead to neuroinflammation, further contributing to cognitive impairment^11^.

Cognitive function demonstrates a complex pattern of change following heart transplantation. In the immediate postoperative period, some patients may experience an initial improvement in cognitive function. This improvement could be attributed to the increase in cardiac output and subsequent enhancement of cerebral perfusion^12^. The improved hemodynamic status can lead to better oxygenation and perfusion of the brain, positively influencing cognitive performance^12,13^. However, this improvement is not universal. Some patients may experience transient cognitive impairment in the early postoperative period because of reasons such as anesthesia, surgical stress, and the effects of medications^13^. Transient cognitive impairment may also result from the acute stress response and inflammatory cascade following surgery^13^.

The long-term cognitive trajectory after heart transplantation is more complex. While some studies have reported sustained cognitive improvement, others have shown persistent or progressive cognitive decline. Longitudinal studies have suggested that a substantial proportion of heart transplant recipients experience cognitive decline in the long term, particularly affecting executive function and memory^13,14^. The chronic effects of immunosuppressive medications, vascular alterations, and other contributing factors most likely influence these late-onset cognitive changes^15^. Additionally, psychosocial factors, including recipient beliefs about donors, may indirectly influence self-reported experiences, potentially including cognitive changes, although further research is needed to understand this effect fully^18^. The complex interplay of these factors creates a heterogeneous picture of cognitive outcomes post-transplant.

### 2.2. Contributing factors to neurocognitive changes

Several factors contribute to the neurocognitive changes observed following heart transplantation:

• Immunosuppressive medications, which are essential for preventing graft rejection, can have significant neurotoxic effects. Calcineurin inhibitors, such as cyclosporine and tacrolimus, are frequently associated with an increased risk of cognitive impairment, tremors, and seizures^15^. These medications can potentially disrupt neurotransmitter function and induce structural changes in the brain, contributing to cognitive deficits. The complex interplay between these medications, their dosages, and patient-specific responses makes it challenging to isolate the specific contributions of each drug to neurocognitive outcomes^15^. Other medications administered post-transplant, such as corticosteroids and antihypertensive drugs, can also contribute to neurocognitive side effects, further complicating the clinical picture^15,16^.
• Vascular and metabolic factors also play a vital role in post-transplant cognitive outcomes. Hypertension, diabetes, and hyperlipidemia, which are common comorbidities in heart transplant patients, can contribute to cerebral small vessel disease and subsequent cognitive decline^16^. These conditions can lead to microvascular brain injury, impairing cerebral blood flow and contributing to cognitive dysfunction. Changes in cerebral blood flow, vascular remodeling, and inflammation can also influence cognitive function. As the brain relies on a constant blood supply, any interference with this supply will lead to interference with cognitive function. The cumulative impacts of these vascular and metabolic factors over time can lead to progressive cognitive impairment^16^.
• Psychological and emotional stressors, such as the anxiety and depression associated with chronic illness and the transplant process, can exacerbate cognitive deficits. The emotional stress of waiting for a transplant, undergoing major surgery, and dealing with a chronic condition can significantly impact cognitive function. Furthermore, rejection anxiety and the need for lifelong medical management can create chronic stress, further contributing to cognitive decline^17^. The interplay of physical and psychological stressors can profoundly impact neurocognitive outcomes. Additionally, there is the potential for personality changes following heart transplantation, and despite controversial mechanisms, cellular memory has been suggested as playing a potential role^19^. This potential change in personality and the associated stress could also influence cognitive outcomes, adding a further element of complexity to post-transplant neurocognitive function.

## 3. The Controversy of Memory and Behavioral Transfer

### 3.1. Anecdotal reports and case studies

The idea that organ transplantation could result in transferring memories, preferences, or personality traits from the donor to the recipient is a topic that blurs the line between science and speculation. While no conclusive scientific evidence supports this phenomenon, anecdotal reports and case studies have fueled the debate^8^. Numerous documented cases have described transplant recipients experiencing changes in personality, preferences, or memories that seemingly align with those of their donors^8,18,20,21^. These reports often include vivid details that are difficult to explain through conventional medical or psychological frameworks^28^ (Table 1).

**Table 1:** Summary of anecdotal reports of behavioral changes post-transplant. [Disclaimer: These uncontrolled case reports represent unverified patient anecdotes lacking scientific validation. They are presented solely to illustrate reported phenomena requiring rigorous investigation]

**Case**	**Recipient detail**	**Reported changes**	**Possible explanation**	**Source**
1	47-year-old male	Sudden love for classical music; donor was a violinist.	Psychological influence or coincidence.	Bunzel et al., 1992^8^
2	29-year-old female	Developed a fear of dark places; donor died in a car crash at night.	Emotional transfer or psychosomatic response.	Pearsall et al., 2002^7^
3	56-year-old male	Began painting landscapes; donor was an artist.	Placebo effect or identity integration.	Pearsall et al., 2002^7^
4	34-year-old female	Craved beer and spicy foods; donor was a beer enthusiast.	Psychological association or cellular memory.	Pearsall et al., 2002^7^
5	62-year-old male	Developed a passion for motorcycles; donor was a biker.	Suggestibility or subconscious adoption of donor’s traits.	Mauthner et al., 2015^27^
6	12-year-old girl	Started speaking Spanish fluently; donor was a native Spanish speaker.	Psychological influence or subconscious learning.	Anthony et al., 2018^58^
7	50-year-old male	Experienced vivid dreams of flying; donor was a pilot.	Subconscious processing of donor’s memories.	Pearsall et al., 2002^7^
8	41-year-old female	Developed a fear of water; donor drowned.	Emotional transfer or psychosomatic response.	Pearsall et al., 2002^7^

One of the most famous cases involves Claire Sylvia, a heart-lung transplant recipient who documented her experiences in the book “A Change of Heart”. Sylvia reported developing new preferences for foods, such as chicken nuggets and green peppers, which she later discovered were favorites of her donor. She also described experiencing vivid dreams and emotional changes that she attributed to the donor’s personality^22^. Another case involved an 8-year-old heart transplant recipient who began having recurring nightmares about being killed by a man. The child’s mother later learned that the donor had been murdered, and the details of the nightmares closely matched the circumstances of the donor’s death^23^.

While these cases are often dismissed as coincidental or psychosomatic, some patterns have emerged. Recipients frequently report changes in food preferences, emotional responses, and hobbies that align with the donor’s known characteristics. These changes are often accompanied by a sense of “otherness”, as if the recipient has incorporated aspects of the donor’s identity^27^. Some other reported cases are summarized below:

The medical community—surgical and psychological researchers—remains divided on the possibility of memory and behavioral transfer. Some schools of thought propose that cellular memory, which suggests that memories and traits could be stored in cells outside the brain, might explain these phenomena. However, this theory lacks empirical support and is often criticized for being speculative^7,23,24^.

Others argue that the reported changes can be explained by psychological factors, such as the recipient’s awareness of the donor’s identity or the profound emotional impact of receiving a life-saving organ. The placebo effect, suggestibility, and the brain’s ability to create narratives to make sense of traumatic experiences may also play a role ^24–26^.

The controversy surrounding memory and behavioral transfer in heart transplant recipients highlights the complex interplay between biology, psychology, and identity^29^. While anecdotal reports and case studies provide intriguing insights, they fall short of offering definitive evidence. Further research, including longitudinal studies and neuroimaging analyses, is needed to explore these phenomena systematically. Until then, the question of whether a donor’s memories and traits can influence a recipient remains one of the most fascinating mysteries in transplantation medicine.

### 3.2. Theories explaining memory/behavioral transfer

It was previously thought that the heart was solely responsible for preserving life functions by pumping oxygenated blood to the brain and the rest of the body; however, the rise of a curious phenomenon seen mostly in heart transplant recipients, where behavioral changes, personality changes, and memory flashes mimicked those of their donors in specific circumstances^19^, has opened a door to new theories that might unlock our understanding of memory and personality.

Numerous hypotheses have been proposed to explain this observed personality change following organ transplantation. These hypotheses can be grouped into three categories: biochemical, psychological, and electrical/energetic (Table 2).

**Table 2:** A summary of influential hypotheses and proposed theories.

**Hypothesis type**	**Resultant Theory**	**Theory position**
*Biochemical Hypotheses*	Cardiac Neurological memory theory [heart brain theory]	Proposes that the heart and brain interact bidirectionally, influencing memory, emotions, and cognition beyond the heart’s mechanical role^30^
	Engram Theory	Posits that memories are stored as physical or biochemical traces (engrams) in neural networks, formed through synaptic changes, enabling memory recall and learning^31^.
	Cellular memory Theory	Proposes that cells outside the brain (e.g., organs) retain memories or experiences through biochemical/epigenetic changes^32^.
*Psychological Hypotheses*	Donor Fantasy Theory	Examines how donors’ idealized visions of their impact, rather than actual outcomes, motivate philanthropic acts and fulfill personal or emotional desires^40^.
	Magical/Analogy Thinking Theory	Explains how humans use symbolic parallels or supernatural associations (analogies) to interpret reality, often bypassing logic for intuitive or cultural connections^29^.
*Electrical/Energy Hypotheses*	Electromagnetic Field Transfer Theory	Posits that electromagnetic fields influence neural pathways, potentially altering behaviors or ”transferring” learned patterns^40^.

#### 3.2.1. Biochemical hypothesis

The biochemical hypotheses are centered on the concept that the donor organ has the ability to store memories which are then transferred to the organ recipient. Theories behind this include Cardiac Neurological Memory—the heart, often referred to as the “second brain”, harbors an intricate network of neurons and neurotransmitters, which play a vital role in regulating cardiac function and may also be involved in the encoding and storing of memories^30^; the Engram Theory, which postulates that engrams are formed in the brain of the donor and these engrams are transferred to the brain of the recipient via exosomes^31^; and the Cellular Memory Theory^19,29^, which is a particularly intriguing concept that suggests memory can be encoded within the cells of an organ via four pathways: the epigenetic/DNA, non-coding RNA, and protein memory storage pathways^32^.

Epigenetic modifications, which involve chemical alterations to DNA and histone proteins, can influence gene expression and have been implicated in the storage and transmission of information across generations. Their existence suggests that experiences and environmental factors can leave lasting imprints on an individual’s genome, potentially influencing their behavior and personality. This form of memory, encoded in the epigenome, could theoretically be passed on to the recipient following a heart transplant^33–36^.

Also, many studies have demonstrated the transfer of long-term memory between individuals by injecting RNA extracted from trained donors into naive recipients. This suggests that RNA-mediated mechanisms implicated in various biological processes, including memory formation and synaptic plasticity, could contribute to transmitting memories or behavioral traits from heart donors to recipients^32,37^.

Protein memory storage, made evident by the presence of prions in exosomes, as prions have since been implicated in normal physiological processes, including synaptic plasticity and memory formation, raises the possibility that these particles could also serve as vehicles for transferring memory-related molecules between cells^38,39^.

#### 3.2.2. Psychological hypotheses

These revolve around the idea that the perceived changes in recipient behavior and personality result from fantasies about the donor and donor’s organ^40^, or defense mechanisms employed to deal with the stress of surgery^7^. Magical/analogical thinking postulates that since a mixed substance shows traits from all the components that comprise that substance, one could expect the same could occur following transplantation where people view themselves as a mix of their own body and the organ of the donor^29^.

#### 3.2.3. Electrical/energy hypothesis

While this hypothesis remains speculative, it underscores the interconnectedness of physiological processes, highlighting that energy and information are fundamentally related and since the heart generates the largest electromagnetic field in the body, information related to the personality of the donor could be stored within the electromagnetic field of the donor’s heart and this information could then be transferred with the heart to the recipient^40^.

### 3.3. Skepticism and scientific criticism

While this appears as an exciting new insight into our understanding of memory, cellular communication, and biological interconnection, it is important to note that most of these proposed mechanisms remain theories. There is a persistent lack of data and information that can be presented as conclusive evidence, and previous studies investigating personality changes following organ transplantation are limited by small sample sizes, lack of comparison groups, subjective assessments, and a lack of prospective studies. Also, there is little information regarding similar personality changes following the transplantation of other organs, and minimal quantitative evidence exists to determine the frequency of personality changes following the transplantation of any organ^21^.

## 4. Mechanistic Hypotheses and Neuroscientific Perspectives

### 4.1. The role of the heart-brain axis

The heart-brain axis represents a bidirectional communication system integrating neural, biochemical, and biomechanical signals between the cardiovascular and central nervous systems. This intricate interplay is particularly relevant in the context of heart transplantation, where neurocognitive and behavioral changes in recipients have been anecdotally reported^41^. Understanding the mechanistic underpinnings of these phenomena requires exploring the neurophysiological connections of the heart and the role of the autonomic nervous system (ANS) in modulating cognition and emotion.

**Figure 1.**
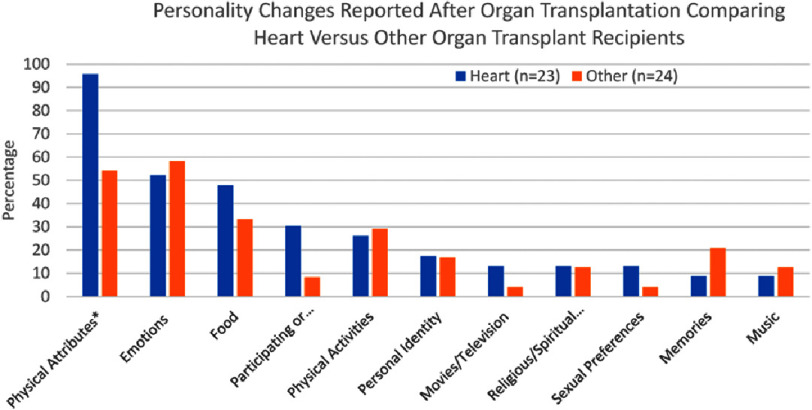
An overview of personality changes following organ transplantation. Personality Changes Associated with Organ Transplants ©  2022 by Carter et al. is licensed under CC BY-NC-ND 4.0. To view a copy of this license, visit https://creativecommons.org/licenses/by-nc-nd/4.0/.

Figure 1 is a graphical illustration of personality changes following organ transplant^18^:

#### 4.1.1. The heart’s neurophysiological connections

Historically, the heart has been regarded as a mere pump facilitating circulation, yet emerging evidence suggests a more dynamic role in neurophysiology. The heart contains an intrinsic cardiac nervous system (ICNS), often referred to as the “heart’s little brain”, composed of a dense network of ganglia, neurons, and neurotransmitters capable of modulating cardiac function independently of central oversight^42^. This ICNS communicates extensively with the central nervous system (CNS) via afferent and efferent pathways, primarily mediated by the vagus nerve and sympathetic fibers.

Cardiac afferent fibers project to key brain regions, including the medulla oblongata, hypothalamus, and limbic structures such as the amygdala and hippocampus. These areas are integral to emotional regulation, memory processing, and cognitive function. Through neuroplastic mechanisms, disruptions or modifications in cardiac neural input—such as those resulting from heart transplantation—may influence these brain regions, potentially leading to altered neurocognitive and behavioral patterns in recipients^43^.

Furthermore, neurotransmitters such as norepinephrine, dopamine, and serotonin, which are crucial in modulating mood and cognition, are also produced and regulated within the cardiac environment. Changes in their homeostasis due to denervation and subsequent reinnervation following transplantation could contribute to post-transplant neuropsychological alterations. Additionally, hormonal changes associated with cardiac function, such as atrial natriuretic peptide (ANP) release, have been implicated in influencing stress responses and cognitive processing. The interplay of these biochemical signals highlights the broader systemic effects of cardiac transplantation on neural mechanisms.

#### 4.1.2. Influence of the autonomic nervous system on cognition and emotion

The ANS, comprising sympathetic and parasympathetic branches, is pivotal in regulating cardiovascular function, stress responses, and emotional states. The vagus nerve, a major component of the parasympathetic system, facilitates communication between the heart and the brain, influencing heart rate variability (HRV), a biomarker associated with emotional resilience, executive function, and stress adaptation.

Post-transplant, the heart is initially denervated, severing direct neural communication between the donor heart and the recipient’s CNS. Over time, partial reinnervation occurs, predominantly via sympathetic fibers, while parasympathetic reinnervation remains limited^44^. This altered autonomic balance may contribute to cognitive and emotional shifts in transplant recipients, potentially explaining reports of behavioral changes and memory phenomena linked to donor characteristics.

Moreover, ANS dysfunction has been implicated in various neuropsychiatric disorders, including depression, anxiety, and cognitive decline. Given that heart transplant recipients often experience ANS instability during the reinnervation process, they may be more susceptible to neurocognitive fluctuations^45^. The altered autonomic tone could disrupt the regulation of limbic structures, thereby influencing emotional processing and personality traits. Additionally, stress-induced changes in autonomic function may exacerbate cognitive disturbances, making post-transplant monitoring and rehabilitation critical for improving patient outcomes.

The heart-brain axis serves as a crucial interface between cardiovascular function and neurocognition, with profound implications in heart transplantation. The neurophysiological connections of the heart, coupled with the regulatory role of the ANS, provide a plausible framework to investigate reported changes in memory and behavior among transplant recipients. Further interdisciplinary research integrating cardiology, neuroscience, and psychophysiology is essential to elucidate the underlying mechanisms and their implications for post-transplant care. Understanding these interactions will enhance transplant outcomes and offer insights into novel therapeutic approaches for managing cognitive and emotional health in transplant recipients.

### 4.2. Post-heart transplantation: investigating neurocognitive outcomes and the dilemma of memory and behavioral transfer between donor and recipient

#### 4.2.1. Potential epigenetic changes in transplant recipients

Studies indicate that heart transplant recipients may exhibit preferences, emotions, and memories resembling those of the donors, suggesting a form of memory storage within the transplanted organ. A mechanism proposed for this memory transfer is epigenetic modifications^32^.

Epigenetic modifications, which involve chemical alterations to DNA and histone proteins, can influence gene expression and have been implicated in the storage and transmission of information^46^. While electrocardiography may reveal alterations post-transplantation, emotional imprints may transfer to recipients, potentially influencing their psyche. Genetic composition, notably RNA and DNA sequences in cardiomyocytes, may perpetuate such memory, impacting recipients’ emotional experiences^47^.

Prions have been implicated in normal physiological processes, including synaptic plasticity and memory formation. The presence of prions in exosomes raises the possibility that these proteinaceous particles could serve as vehicles for transferring memory-related molecules between cells^48^. The existence of epigenetic memory suggests that experiences and environmental factors can leave lasting imprints on an individual’s genome, shaping not only their phenotype but also potentially influencing aspects of their behavior and personality. This form of memory, encoded within the epigenome, provides a mechanism through which the experiences of the donor could theoretically be passed on to the recipient following a heart transplant^36^.

#### 4.2.2. Immune system’s influence on brain function and behavior

The brain, endocrine, and immune systems are inextricably linked. Immune molecules have a powerful impact on neuroendocrine function, including hormone-behavior interactions, during health and sickness^49^.

Psychoneuroimmunology has demonstrated an intricate network of bidirectional relationships between the immune system and the brain. Pro-inflammatory cytokines, including interleukin (IL)-1, IL-6, and tumor necrosis factor (TNF)-alpha, are released by activated immune cells during psychosocial stress. These factors represent primary mediators of the communication between the immune system and the brain, and coordinate the behavioral changes necessary for recovery^50^.

Behavioral consequences of these effects of the immune system on the brain include depression, anxiety, fatigue, psychomotor slowing, anorexia, cognitive dysfunction, and sleep impairment^51^.

## 5. Psychological Adaptation in Transplant Recipients

The acquisition of donor personality characteristics by recipients following heart transplantation is hypothesized to occur via the transfer of cellular memory, and four types of cellular memory are presented: epigenetic memory, DNA memory, RNA memory, and protein memory. Other possibilities, such as the transfer of memory via intracardiac neurological memory and energetic memory, are also discussed^19^.

Pearsall et al. interviewed 10 heart transplant recipients and found that each had 2 to 5 characteristics similar to those of their respective donors^7^. Similarly, Mauthner et al. reported on 25 transplant recipients, of which 92% mentioned changes in self-identity and perception, 36% felt that the donor was still alive through the transplanted hearts, and 56% mentioned that they had dreamed about or guessed the donor’s appearance^52,53^. Additional etiologies, other than organic causes, such as abnormal serum tacrolimus levels, have been proposed to explain the changes in affective disturbances and elusive delusions observed in heart transplant patients^54^. Liester proposed that donors’ emotions and temperament may be transferred through heart transplantation via cell signaling mechanisms, such as genetic or epigenetic pathways, the intracardiac nervous system, or the heart’s electromagnetic energy^19^.

### 5.1. Post transplant psychological distress and coping mechanisms

After transplantation, the psychosocial burden is usually less severe than during the preoperative period. Nevertheless, patients must still be regarded as chronically ill and have to demonstrate considerable coping skills. Unsuccessful readjustment is associated with lower QOL and psychiatric morbidity^55,56^. The most common psychological disorders among patients before and after transplantation are affective and anxiety disorders^57^ (Table 3).

**Table 3:** Summary of post-transplant psychological disorders and their coping mechanisms.

Psychological Distress	Coping Mechanism
Postoperative delirium [acute organic brain syndrome]	Facilitate processing of delirium and psychotic symptoms
Traumatic Experiences, fear of rejection and coping with complications	Facilitate processing of traumatic reactions, fear and pain Support/ encouragement in case of somatic crisis
Fear of organ failure	Facilitate fear processing and emotional stabilization
Fear of infections and other comorbidities [eg cancer]	Facilitate adherence and readjustment Crisis intervention
Coping with medical problems	Supportive therapy
Readjustment and adherence problems	Cognitive and behavioural interventions
Hopelessness, depression, feelings of guilt, increased fear of retransplantation	Mediate contact to patient support group

### 5.2. Ethical Implications of Memory Transfer Claims

Addressing ethical implications has been of increasing concern since the rise of heart transplantation in the 20th century^32^. These issues stem from the dynamic changes in defining brain death in the donors, addressing potential personality changes in the recipients, and the cellular memory hypothesis^19^.

#### 5.2.1. Medical ethics in discussing anecdotal cases

The reports of changes in preferences regarding music, emotions, and sex are increasingly important, necessitating ethical considerations for organ donors and recipients^19^. Evidence supporting heart memory is advancing with technological improvements, providing insights into the concepts of multiple memories beyond the brain^18,32^. Although there is a paucity of studies that have investigated memory transfer claims, there are reports of such cases, including a 45-year-old male recipient from a 17-year-old donor who reported the need to use earphones and listen to loud music post-transplantation^19^. Cases like the foregoing example underscore the need for robust informed consent detailing a potential memory transfer post-transplantation^25^.

#### 5.2.2. Implications for donor families and recipients

The neurocognitive outcomes following heart transplantation significantly influence emotional and psychological well-being^32^. Additionally, alterations in recipients’ behavior may induce either distress or appeal among their family members^25^. Findings from analogous studies indicate a pronounced correlation between the food preferences of recipients and those of their donors, as confirmed by the families of the donors^25^. Moreover, significant changes in temperament, emotions, and unique dream-related experiences have been observed^25^. These transformations necessitate establishing a support system for donors and their families to facilitate the navigation of these experiences while adapting to their new realities^25^.

### 5.3. Ethical Implications of Memory Transfer Narratives

The dissemination and clinical discussion of speculative theories, such as memory or personality transfer from donor to recipient, present significant ethical challenges in the context of cardiac transplantation. While these narratives may seem benign or even comforting to some, promoting unsubstantiated claims can inadvertently cause psychological harm, distort public understanding, and undermine the therapeutic alliance between patients and healthcare professionals.

#### 5.3.1. Potential Risks and Harms

**Psychological Distress in Recipients**: Heart transplant recipients may experience heightened anxiety, identity confusion, or survivor’s guilt if they perceive themselves to have “inherited” the traits, emotions, or traumas of their donors. This can complicate recovery, reduce treatment adherence, and trigger maladaptive coping behaviors^19,25^.

**Distorted Family Expectations and Grief Responses**: Donor families may seek symbolic continuities between their deceased loved ones and recipients, interpreting behavioral similarities as proof of ongoing presence. While emotionally compelling, such beliefs can delay the healthy processing of grief and introduce unrealistic expectations for the transplant outcome^25,27^.

**Misinformation and Public Misperception**: If speculative theories are presented without appropriate scientific context or caution, they risk sensationalizing transplantation, fueling media distortion, and undermining trust in the medical community—particularly when later debunked or contradicted by emerging evidence^7,19^.

**Consent and Disclosure Complexities**: Discussing speculative memory transfer narratives during pre-transplant counseling or informed consent processes may confuse patients and complicate risk-benefit assessments, especially when patients already face emotional vulnerability^25,32^.

#### 5.3.2. Clinical Recommendations

To uphold ethical standards of care, transplant teams and clinicians should adopt the following principles when navigating this sensitive topic:

**Prioritize scientific accuracy and transparency**: Clearly differentiate between evidence-based neurocognitive outcomes (e.g., effects of surgery, immunosuppressants, or cerebral perfusion changes) and anecdotal or speculative phenomena. Avoid implying causality where none exists^3,5,15^.

**Frame identity changes in multifactorial contexts**: Normalize emotional and psychological responses as part of the transplant experience—shaped by surgical stress, chronic illness, medication side effects, and the existential weight of receiving an organ—not as mystical donor traits^10,17,54^.

**Avoid validation of unproven theories**: Refrain from endorsing or promoting cellular memory, donor “soul” transference, or similar metaphysical explanations unless clearly labeled as unsubstantiated hypotheses^7,9,19^.

**Provide tailored psychological support**: Recognize that some patients may struggle with self-concept or experience existential dilemmas post-transplant. Offer access to mental health professionals skilled in transplant psychology, identity formation, and trauma-informed care^17,56,57^.

**Develop ethical counseling protocols**: Incorporate training for clinicians on how to sensitively respond to memory transfer narratives without dismissing the patient’s lived experience or encouraging scientifically unsupported beliefs^19,25,52^.

**Foster interdisciplinary dialogue**: Collaborate with ethicists, psychologists, transplant surgeons, and spiritual care providers to develop balanced counseling frameworks that respect patient narratives while grounding discussions in evidence-based medicine^10,55^.

## 6. Research gaps and prospects

**Longitudinal neurocognitive studies**: Existing literature lacks robust longitudinal data mapping pre- to post-transplant cognitive trajectories. Prospective studies tracking neuropsychological performance, epigenetic profiles, and psychosocial adaptation over extended periods are essential to identify causality between transplantation and cognitive shifts, distinguishing these from age-related or medication-induced changes.

**Mechanistic validation of cellular memory**: While hypotheses such as epigenetic memory, RNA transfer, and neurocardiac signaling are theorized, empirical validation is scarce. Controlled experiments using animal models, coupled with advanced neuroimaging (e.g., fMRI, PET scans) and biomarker analyses, could delineate the role of donor-derived cells in modulating recipient cognition.

**Psychosocial and ethical frameworks**: The ethical implications of reported identity changes—including informed consent processes and donor-recipient confidentiality—remain underexplored. Standardized protocols for documenting anecdotal claims, alongside culturally sensitive support systems for recipients and donor families, are urgently needed.

**Intervention development**: Research must evaluate targeted interventions for recipients experiencing distressing neurocognitive changes. Cognitive rehabilitation programs, pharmacogenomic tailoring of immunosuppressants, and psychotherapeutic modalities require clinical trials to assess efficacy.

**Cross-organ comparisons**: Investigations comparing neurocognitive outcomes across organ transplant types (e.g., liver, kidney) could clarify whether observed phenomena are heart-specific or systemic, informing broader biological-psychological models.

## 7. Limitations and alternative explanations

This review is limited by heterogeneity in neurocognitive assessments across studies. Crucially, evidence for memory transfer derives solely from uncontrolled anecdotes. Rigorous prospective studies controlling for medication effects, suggestion, and reporter bias are absent. The ethical risks of promoting unvalidated theories to vulnerable patients warrant caution. Despite the compelling narratives surrounding memory and behavioral changes post-transplant, several limitations compromise the validity of these claims:

• **Methodological Constraints**: Most evidence for personality or memory transfer is derived from uncontrolled anecdotal case reports lacking blinding or control groups. No large-scale longitudinal or prospective studies exist to validate these observations or identify confounding variables.
• **Confirmation Bias and Suggestibility**: Recipients aware of their donor’s identity may unconsciously attribute behavioral changes to the donor. Confirmation bias likely influences both patient perception and the clinician’s interpretation of post-transplant behaviors. Highly emotional contexts of transplantation increase suggestibility and the possibility of constructed memories.
• **Placebo and Psychological Adaptation**: Recipients often undergo a profound psychological adjustment, including changes in self-concept, which could be misinterpreted as memory transfer. The placebo effect and coping mechanisms may result in behaviors mistakenly linked to donor traits.
• **Coincidental Similarities**: Statistical probability dictates that some behavioral overlaps between donor and recipient will occur purely by chance, especially in small sample anecdotes.
• **Neurotoxicity and Pharmacologic Effects**: Immunosuppressants, especially calcineurin inhibitors, are neurotoxic and known to affect mood, cognition, and perception. These side effects may mimic symptoms interpreted as donor-related behavioral changes.
• **Inadequate Theoretical Validation**: The cellular memory hypothesis lacks experimental confirmation. Theories involving electromagnetic fields, RNA-mediated engrams, or epigenetic imprinting remain speculative without rigorous scientific support.

## 8. Conclusion

Heart transplantation transcends physiological restoration, invoking profound neurocognitive and psychological complexities in recipients. While anecdotal accounts of memory and behavioral transfer captivate scientific and public interest, they remain contentious due to methodological limitations and the absence of mechanistic evidence. Current data suggest that neurocognitive changes arise multifactorially, mediated by immunosuppressive neurotoxicity, vascular comorbidities, and psychosocial stressors. The hypothesis of cellular memory, though provocative, demands rigorous validation through interdisciplinary collaboration.

Clinically, prioritizing neuropsychological monitoring and tailored psychosocial support is paramount to mitigating post-transplant cognitive decline and identity-related distress. Ethically, transparent dialogue with recipients about unverified phenomena must balance hope with scientific caution. Future research integrating longitudinal neuroimaging, epigenomic profiling, and cross-disciplinary frameworks will clarify these enigmas, advancing transplantation medicine beyond survival metrics to holistic patient well-being.

## Declarations

Ethics approval and consent to participate: Not applicable

Consent for publication: Not applicable

Availability of data and material: Not applicable

## Competing interests

We declare NO competing interests

## Funding

Not applicable

## Authors’ contributions

P.A conceptualised the study, analysed the data, wrote the initial draft, validated the final draft and supervised the execution of the manuscript. ALL authors co-wrote the initial draft, contributed to the final draft and reviewed the manuscript. All authors contributed to the manuscript, read and approved the final manuscript.

## Acknowledgements

The authors would like to acknowledge THE LIND LEAGUE, Nigeria for providing the invaluable resources to kick start, culminate and leverage this research project while also enabling our capacities.
