# Correction to “Neurocognitive outcomes and memory transfer in heart transplantation”

**DOI:** 10.21542/gcsp.2026.19

**Published:** 2026-04-30

Dear Editor,

We are writing regarding our manuscript, “Neurocognitive outcomes and memory transfer in heart transplantation”, which was published in issue 4 (2025) of *Global Cardiology Science and Practice*^1^.

Following a detailed review of our manuscript, we have identified two errors that we wish to correct.

## A. Correction to Citation

We have identified that citation number [23] on page 7 of our manuscript is incorrect. The reference currently cited in this position is inaccurate and should be replaced.

The correct reference for citation [23] is as follows:

Esposito, J. (2024, November 29). *Little girl who received heart transplant solved murder of her donor?* Snopes. [Retrieved 15 Jan 2026] from https://www.snopes.com/fact-check/girl-solved-murder-heart-transplant-donor/.

## B. Incorrect Table Entry

Row 6 from Table 1. Upon further review, we have determined that this entry, which concerns a 12-year-old girl in a Spanish language case, contains inconsistencies and potential inaccuracies. To ensure the reliability of the published data, we request that the entirity of row 6—including its text and the corresponding reference—be ignored.

Please do not hesitate to contact us if you require any further information or clarification.

Sincerely,

Patrick Ashinze.

## References

[1] Ashinze P, Salawu W, Akande E, Banerjee S, Idris-Agbabiaka A, Chukwu B, Mafua N, Ngirigwa F, Okeoyo T, Olowookere S, Adeleye VM, Olajuwon JT, Aboderin C, Olasemo A, Musa LA (2025). Neurocognitive outcomes and memory transfer in heart transplantation. Global Cardiology Science and Practice.

